# Digital Interventions for Psychological Well-being in University Students: Systematic Review and Meta-analysis

**DOI:** 10.2196/39686

**Published:** 2022-09-28

**Authors:** Madeleine Ferrari, Stephanie Allan, Chelsea Arnold, Dina Eleftheriadis, Mario Alvarez-Jimenez, Andrew Gumley, John F Gleeson

**Affiliations:** 1 Healthy Brain and Mind Research Centre Australian Catholic University Sydney Australia; 2 School of Health and Wellbeing University of Glasgow Glasgow United Kingdom; 3 Healthy Brain and Mind Research Centre Australian Catholic University Melbourne Australia; 4 Centre for Youth Mental Health The University of Melbourne Melbourne Australia; 5 Orygen Melbourne Australia

**Keywords:** psychological well-being, mental health, university students, higher education students, college students, digital intervention, web-based intervention, mobile phone, meta-analysis, systematic review

## Abstract

**Background:**

Life at university provides important opportunities for personal growth; however, this developmental phase also coincides with the peak period of risk for the onset of mental health disorders. In addition, specific university lifestyle factors, including impaired sleep and academic and financial stress, are known to exacerbate psychological distress in students. As a result, university students have been identified as a vulnerable population who often experience significant barriers to accessing psychological treatment. Digital psychological interventions are emerging as a promising solution for this population, but their effectiveness remains unclear.

**Objective:**

This systematic review and meta-analysis aimed to assess digital interventions targeting psychological well-being among university students.

**Methods:**

Database searches were conducted on December 2, 2021, via Embase, MEDLINE, PsycINFO, and Web of Science.

**Results:**

A total of 13 eligible studies were identified, 10 (77%) of which were included in the meta-analysis. Mean pre-post effect sizes indicated that such interventions led to small and significant improvement in psychological well-being (Hedges *g*=0.32, 95% CI 0.23-0.4; *P*<.001). These effects remained, albeit smaller, when studies that included a wait-list control group were excluded (Hedges *g*=0.22, 95% CI 0.08-0.35; *P*=.002). An analysis of acceptance and commitment therapy approaches revealed small and significant effects (*k*=6; Hedges *g*=0.35, 95% CI 0.25-0.45; *P*<.001).

**Conclusions:**

Digital psychological interventions hold considerable promise for university students, although features that optimize service delivery and outcomes require further assessment.

**Trial Registration:**

PROSPERO CRD42020196654; https:/www.crd.york.ac.uk/prospero/display_record.php?RecordID=196654

## Introduction

### The Changing Landscape of Mental Well-being in University Students

Life at university provides important opportunities for personal growth during emerging adulthood by fostering autonomy, increasing social connectedness, and expanding intellectual horizons [1]. However, this developmental phase of emerging adulthood also coincides with the peak period of risk for onset of mental health disorders [2,3], and specific university lifestyle factors, including impaired sleep [4] and academic and financial stress, are known to exacerbate psychological distress in students [5,6].

Such risks are likely to contribute to the findings of the World Mental Health Surveys—International College Student Project by the World Health Organization, which indicates that between 20% and 31% of students experience a diagnoseable mental disorder [7,8]. Mental health symptoms at university entry are known to persist for at least the first year of study [6]. Furthermore, students with mental health disorders have been shown to eventually drop out from their studies at 2.5 times the rate of matched controls [9]. As the prevalence of mental health problems in young people has been steadily increasing [10], university counseling services have been unable to keep up with the growing demand; only 16% of students surveyed in the World Health Organization surveys with mental disorders reported receiving minimally adequate treatment [7].

### Reforming University Counseling and Mental Health Services

There have been recent calls for reform of university mental health services to address the increased prevalence of mental health problems among higher education students [11]. Duffy et al [11] proposed an integrated model of university-based mental health care that embeds multidisciplinary mental health teams within campus health services to provide timely care and to facilitate the transition from and to surrounding services. A significant challenge is that university students have a very broad spectrum of mental health needs, potentially ranging from thwarted personal growth in a narrow domain at one end of the spectrum (eg, suboptimal study habits) to enduring complex psychiatric disorders at the other end (eg, recurrent severe major depression combined with substance misuse). Intermediate needs may include mild and transient mental health symptoms (eg, self-limiting depressed mood) and incipient mental health episodes. There is also evidence that students with serious mental health problems have delayed access to mental health services, as evidenced by longer durations of untreated symptoms of psychosis [12]. The clinical staging model of mental health disorders by McGorryet al [13], which maps mental health interventions against commensurate levels of severity of mental health problems, was incorporated into the model by Duffy et al [11] as a guiding heuristic for responding to this complex spectrum. Specifically, the staging model highlights that the students presenting to university services range from those at early stages of risk with nonspecific mental health symptoms that do not meet criteria for a categorical diagnosis but who require monitoring and support to those at stage 3 and beyond who are recovering from a full threshold mental health disorder. This diversity of presenting problems poses a challenge in determining the effectiveness of mental health interventions for university students.

In the context of this diversity, psychological well-being (PWB) has emerged as an important construct with strong developmental face validity which should be considered when evaluating the effectiveness of university-based mental health interventions. PWB has been defined in terms of specific components of personal growth (including purpose in life, mastery, and self-acceptance) [14] and by the extent to which psychological needs, namely, autonomy, competence, and connectedness, have been met or thwarted (eg, by mental health symptoms) [15]. PWB also offers the advantage of being strongly correlated with psychopathology in youth but is also a distinct construct that may provide a basis for the prevention of mental health problems [16]. In addition, PWB has been identified as having strong transdiagnostic utility in predicting broad psychological outcomes across diverse populations [17,18]. Therefore, it is not surprising that PWB has emerged as a high priority for investigation and intervention in university students [19].

### The Role of Digital Technology in Mental Health Reform

A psychological intervention that seeks to improve mental health outcomes is considered digital when technology is used in its delivery, including the internet, mobile phones, computers, or other electronic devices. Digital interventions for mental health problems of varying severity have rapidly emerged as an innovation that promises improved access, acceptability, scalability, and cost-effectiveness compared with traditional face-to-face services alone [20]. Digital interventions for university students may circumvent students’ concerns regarding stigma, time constraints, and lack of familiarity with health care systems [21]. In addition, high rates of access to smartphones and familiarity with mixed modes of learning mean that most university students are highly amenable to digital modes of health support [21]. Other global factors, such as the COVID-19 pandemic, have further accelerated the implementation of digital interventions as solutions for addressing limitations associated with traditional health care settings for young people [22].

Previous reviews related to this topic have reached contradictory conclusions in relation to PWB. Lattie et al [23] conducted a systematic review investigating the effectiveness, usability, acceptability, uptake, and adoption of digital mental health interventions for university students across a range of outcomes; however, the findings in relation to PWB were not reported separately from other outcomes. Inspection of their supplementary materials showed that across the 89 included publications, 8 studies specifically examined PWB as an outcome. One study measured PWB using a qualitative methodology and found little meaningful improvement, whereas the remaining studies consisted of randomized controlled trials (RCTs) and non-RCT and consistently reported significant improvement using validated measures (RCT: *k*=6; non-RCT: *k*=1). In contrast, a systematic review and meta-analysis of RCTs on the same topic and published in the same year reported 4 RCT studies that explicitly reported a measure of PWB (among other outcomes) when examining a web-based intervention for university students [24]. Although the review reported small and significant improvements in depression and anxiety symptoms, an analysis of the 4 studies that assessed PWB found nonsignificant effects for this outcome [24]. These conflicting results call for a closer examination of the effectiveness of digital interventions for PWB among university students.

There have been recent calls to reform mental health support for university students [11] and to develop stepped care models of psychological intervention to ease demands on existing mental health services [13]. As a result, it is necessary to review the evidence for the effectiveness and quality of digital interventions for PWB in university students. Therefore, the primary aim of this review was to examine the evidence for the effectiveness of digital mental health interventions for university students, specifically in relation to PWB. We also aimed to review the quality of this evidence, and given the diversity of this population, it is especially important to understand the range and severity of mental health symptoms reported in these studies. Our review question, in accordance with the PICO (population, intervention, control, and outcomes) framework [25], was whether web-based digital interventions, compared with active and passive control conditions, improve PWB in university student populations.

## Methods

### Search Strategy and Selection Criteria

The systematic review and meta-analysis were conducted in accordance with the PRISMA (Preferred Reporting Items for Systematic Reviews and Meta-Analyses) statement [25-27]. We conducted a review of trials, including pilot studies, non-RCTs, and RCTs, that evaluated any form of mental health or psychological health intervention targeting university students that was delivered through a web-based or eHealth medium, including mobile phone–based apps. Eligibility criteria included the assessment of a student population from a university or higher education institution (such as a college) of any age or nationality. Eligible studies were also required to be published in peer-reviewed journals in English language during and since 2000. Cohort, case-controlled, and cross-sectional studies were excluded because the study designs did not provide conclusions regarding the effectiveness of the interventions. For inclusion in this review, PWB needed to be assessed as a primary or secondary outcome using a validated measure. PWB is a multifaceted construct, and in this review, it was defined in line with the eudaemonic theories of well-being by Ryff [14] and Ryan et al [28]. Ryff [14] identified 6 core components of PWB that shape healthy development across the life span: self-acceptance, personal growth, purpose in life, environmental mastery, autonomy, and positive relations with others. The model of PWB by Ryff [14] provides a framework for meaning and purpose in life and has been operationalized using measures such as the Psychological Well-Being Scale and the Mental Health Continuum [29]. The self-determination theory by Ryan et al [28] postulates that motivation and wellness can be developed by meeting one’s basic psychological needs: competence, relatedness, and autonomy [15]. The construct of PWB does not include symptoms of distress; thus, measures of distress and psychopathology, such as the depression, anxiety, and stress scale [30], were not included as primary outcome measures for the purpose of the review. The construct of PWB also does not include hedonism; thus, measures of happiness or life satisfaction, such as the Satisfaction with Life Scale [31], were also excluded from this review.

Searches were conducted on December 2, 2021, via Embase (Elsevier), MEDLINE (Ovid), PsycINFO (EBSCOhost), and Web of Science (Thomson Reuters). The search terms used were synonyms for *university student* and *digital intervention* (the full list of search terms and sample search syntax for each individual database is available in Multimedia Appendix 1). We contacted the corresponding authors via email to request further information where clarification of individual studies was required (eg, potential overlap in data across ≥2 published reports). In addition, reference lists of eligible studies and review articles were manually searched. The search was conducted by MF with consultation from a librarian. After deduplication in EndNote X8 (Clarivate Analytics), the completed search was imported into Covidence [32] for screening and data extraction. Abstract and full-text screening was conducted by CA, SA, and MF, with each paper being screened by at least two authors. The interrater reliability for independent full-text screening was reasonable (*k*=0.53; *P=*.76), and after conflicts were resolved by consultation between team members, there was a complete consensus. Data extraction was performed using a template developed by the authors in Excel and undertaken by CA, SA, and JG.

### Data Analysis

The extracted variables included publication characteristics (eg, authors, country, and year of publication), participant characteristics (eg, sample size; mean age; ethnicity; recruitment strategy, and depression, anxiety, and stress symptoms at baseline), study characteristics (eg, study design, primary aim of the study, and treatment conditions), PWB outcome measures (means and SDs at baseline, postintervention, and follow-up time points), and study findings. PWB was the primary outcome of interest. If studies reported multiple follow-up time points, data from the longest follow-up time point after the intervention were extracted.

Risk of bias (RoB) was assessed by CA and JG using the Cochrane collaboration RoB 2 tool [33] for RCTs, and the Downs and Black checklist was used for non-RCTs [34]. In addition, the Grading of Recommendations Assessment, Development, and Evaluation approach was used to evaluate the overall quality of the evidence [35].

We planned to perform a meta-analysis if 3 RCTs enabled the calculation of effect sizes in relation to PWB as a continuous variable. Meta-analysis was performed using Comprehensive Meta-Analysis (version 3), with random effects models used. A sensitivity analysis was performed to ascertain the effect of the wait-list versus active control groups. The *Q* statistic was used to assess study heterogeneity [36]. We also calculated the *I*^2^ statistic to estimate the percentage of variance in the observed effects owing to the variance in the true effects. Heterogeneity can be considered low, moderate, substantial, or considerable, with *I*^2^ values of 0% to 40%, 30% to 60%, 30% to 90%, and 75% to 100%, respectively [37]. The protocol was registered with PROSPERO before screening (registration number: CRD42020196654).

## Results

### Search and Selection

**Figure 1:**
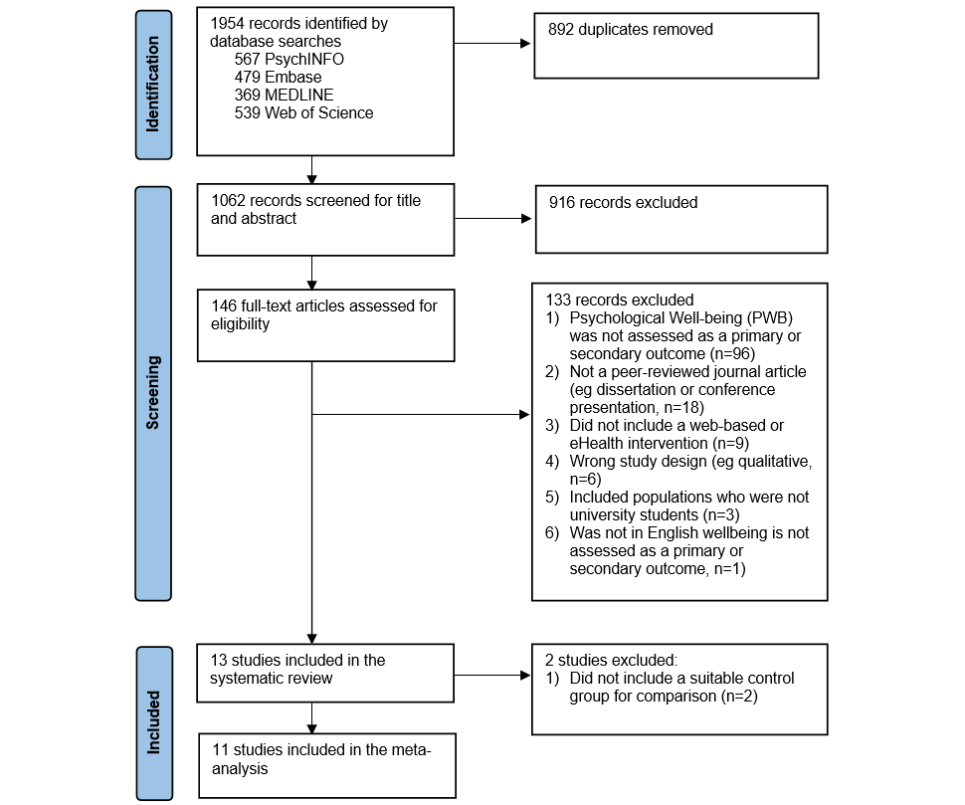
Study selection.

A total of 1954 references were imported into Covidence (Veritas Health Innovation) for initial abstract screening. After removing 892 duplicates, 1062 studies were screened against title and abstract, from which 916 studies were excluded. In all, 146 studies were assessed for full eligibility via full-text screening; the reasons for exclusion are mentioned in Figure 1. Finally, 13 studies met the inclusion criteria and were included in this review [38-50]. Of these 13 studies, 11 (85%) were eligible for inclusion in the meta-analysis [38-42,45,46,48-51].

### Study Characteristics

Table 1 summarizes the characteristics of the included studies. All 13 studies were published from 2016 onward, with 5 (38%) conducted in the United States [39-41,47,49]; 2 (15%) in Sweden [43,48] and Australia [42,44]; and 1 (8%) in Hong Kong [46], Finland [50], Ireland [45], and the United Kingdom [38]. The sample sizes across the 13 studies ranged from 23 [40] to 2110 [44]. Regarding study design, there were 46% (6/13) of RCTs with an active control [39,40,43,45,46,48], 38% (5/13) of RCTs with a wait-list control [38,41,42,49,52], 8% (1/13) were dismantling trials [41], and 8% (1/13) were pre-post trials [47]. The duration of the digital interventions varied, with one study reporting a single session [43], another study reporting 6 days of intervention [44], and the remaining studies (11/13, 85%) ranging from 3 to 10 weeks (mean_duration_=5.5, SD 2.2 weeks; mode duration=4 weeks), as presented in Table 1 [35-42,45-47]. Across all studies, outcomes were measured at pre- and postintervention time points, and the duration of follow-up assessment intervals from baseline ranged from 7 days [46] to 12 months [50].

**Table 1:** Characteristics of studies included in the systematic review.

Study and country	Primary aim	Population		Study features			Intervention			Outcomes and effect size
		N; recruitment; completers (%)^a^	Demographics	Study design	Measurement; time points; PWB^b^ outcome measure	Intervention type; treatment target; delivery mode; duration		Comparison group		
Auyeung et al [46], 2019; Hong Kong	Examine the outcome efficacy of the Best Possible Self intervention in improving well-being	139; university (web-based via university mass mail) and social media; 68.6%	Age: T^c^-22.94 years (SD 3.02; n=48), C^d^-22.7 years (SD 3.68; n=52); female: T-72.9% (n=48), C-73.1% (n=52); ethnicity: NR^e^	Pilot RCT^f^ (T vs active C)	Pre, post; TFS^g^	Best Possible Self Positive Psychology intervention; well-being (increase flourishing and decrease depression); web-based mobile and desktop versions; 6 days		Active: participants wrote about details of 5 events from the past 24 hours	*Flourishing*^h^*:* time × condition effect favoring T. Partial η^2^=0.12; *autonomy:* time x condition effect favoring T. Partial η^2^=0.04; *relatedness:* no time x condition effect. ES^i^ not reported; *conclusion:* intervention more effective at improving flourishing and autonomy compared with active control with moderate effect sizes. No improvements in competence or relatedness	
Bendtsen et al [51], 2020; Sweden	To compare the positive psychology intervention with control	654; 15 universities (digital advertising); 61.2%	Age: T-25 years (median age), C-26 years (median age control); female: T-79.6% (n=277), C-76.1% (n=233); ethnicity: NR	RCT (T C-TAU^j^)	Pre, 3 months after randomization; MHC-SF^k^	mHealth^l^ positive psychology multicomponent program; positive mental health; via smartphone; 10 weeks		Active: web-based mental health information control group. Sent via SMS text message	*Psychological well-being:* group x time effect favoring T. IRR^m^=1.067 (95% CI 1.024 to 1.112); *conclusion:* psychological well-being significantly higher at 3 months in intervention group compared with control group with small effect size	
Firestone et al [47], 2019; United States	Test the usability, acceptability, receptivity, and utility to promote valued living and psychological well-being of the LYV^n^ program	137; university (psychology classes); 84%	Age: 20.22 years (SD 4.35); female: 86.9%; White: 53.3%	Single group: post	Pre, post; SPWB^o^: autonomy subscale	Living Your Values: ACT^p^-based; valued living; web-based, self-guided values-focused program; single 60- to 90-minute session		None	No significant treatment effects (within-group pre-post) on any well-being subscales. ESs not reported; *conclusion:* no evidence that the intervention was effective at improving psychological well-being	
Kvillemo et al [48], 2016; Sweden	Examine the feasibility, usability, acceptability, and outcomes of an 8-week internet-based mindfulness training program	90; university; 39.1%	Age: T completers-29 years (range 18–45 years, n=18), T noncompleters-24 years (range 19-37, n=22); female: T completers-88.9% (n=18) T noncompleters-63.6% (n=14); ethnicity: NR	Pilot RCT (T vs active C)	Pre, post; SPWB: total score	Mindfulness training program; mindfulness; internet-based; 8 weeks		Active: internet-based expressive writing intervention. Participants could make contact with study coordinators via phone or email	*Psychological well-being:* no time x group interaction. T had a statistical increase in psychological well-being over time. Cohen *d*=0.2; *conclusion:* no evidence for group effect in relation to improving well-being. Treatment group showed statistical and small improvement in well-being from preintervention to postintervention	
Levin et al [39], 2016; United States	Test the feasibility of a web-based ACT prototype prevention program called ACT-CL^q^	234; university (digital advertising); 70%	Age: 21.61 years (SD 5.48); female: 76.9%; White: 6.2%, Asian: 9.3%, Black or African American: 3.5%, American Indian or Alaska native: 1.8%, native Hawaiian or other pacific islander: 1.3%, and other: 7.9%	Pilot RCT (T vs active C)	Pre, post, 1-month FU^r^, 3-month FU; MHC-SF	ACT-CL; psychological symptoms, positive mental health, and psychological flexibility; web-based, 2 core multimedia sessions and supplementary emails, web-based resources, and SMS text messages; 3 weeks		Active: 2 session mental health education website (length matched to ACT-CL) basic educational information	*Psychological well-being:* no significant group x time effect. Effect size not reported; *Conclusion:* the effects of the ACT-CL program were largely equivalent to those of an education website, with a lower level of program engagement with ACT-CL	
Levin et al [49], 2017; United States	Evaluate a transdiagnostic web-based self-help program that includes all ACT components and is designed to treat a wide range of problems	79; university (2 cohorts; fall 2014 and spring 2015); 80%	Age: 20.51 years (SD 2.73); female: 66%; White: 88%	Pilot RCT (T v wait-list C)	Pre, post; MHC-SF	ACT; mental health problems; self-help website; 4 weeks		Wait-list	*Positive mental health:* time x condition effect favoring T Cohen *d*=0.58; *emotional well-being:* no significant time x condition interaction. ES not reported; *social well-being:* time x condition effect favoring T Cohen *d*=0.69; *Psychological well-being:* no significant time x condition effect. ES not reported; *Conclusion:* the intervention was not more effective than wait-list control for improving psychological well-being	
Levin et al [40], 2020; United States	Evaluate the feasibility and acceptability of a popular mindfulness meditation app (SBT^s^) for students on a college counseling center wait-list	23; university (counseling service); 60%	Age: 20.43 years (SD 2.46); female: 100%; non-Hispanic White: 87%	Pilot RCT (T v wait-list C)	Pre, during (2 weeks), Post (4 weeks after baseline); MHC-SF	SBT; mindfulness; mobile app; 4 weeks		Wait-list	*Psychological well-being:* statistical effects not calculated owing to small sample size. Pre-post T favored SBT for positive mental health: Hedges *g*=0.52 (95% CI−0.31 to 1.41); *Conclusion:* no evidence for statistical effect on well-being for intervention compared with wait-list control. Small to moderate effect size favoring intervention at postintervention	
Levin et al [41], 2020; United States	Compare web-based versions of ACT targeting the open components, the engaged components, or full ACT, relative to a wait-list condition	181; university; 88.9%	Age: 22.27 (SD 5.08 years); female-72.4%; White: 92.8%	Randomized dismantling trial with 4 conditions (full ACT vs active, open vs active, engaged vs wait-list control)	Pre, post, 4-week FU; MHC-SF	12- session web-based ACT intervention (with differing components); acceptance, cognitive diffusion (open), values, committed action (engaged); via computer or smartphone; 6 weeks		2 Active; open components (open); engaged components (engaged); and wait-list control	*Psychological well-being:* time x condition effect – full or engaged>wait-list (pre-post, pre-FU), wait-list did not differ from open, engaged>open (pre-post). Cohen *d* wait-list vs full=0.51, wait-list vs engaged=0.69, engaged vs open=0.56; *Conclusion:* “Engaged” and “full” intervention, but not “open” intervention more effective at improving positive mental health compared with wait-list with medium effect sizes	
Noone et al [45], 2018; Ireland	Investigate if regular mindfulness meditation practice facilitates critical thinking through the enhancement of executive function	91; university; 72.9%	Age: 20.92 years (SD 4.39); female: 76%; ethnicity: NR	RCT (T v active C)	Pre, post; WEMWBS^t^	Headspace mindfulness; mindfulness; mobile app; 6 weeks		Active: Sham mindfulness app	*Subjective well-being:* group assignment not associated with well-being at FU; b=2.01, 95% CI –0.63 to 4.65; *Conclusion:* no evidence that intervention more effective than active control for improving subjective well-being	
Ponzo et al [38], 2020; United Kingdom	Test the efficacy of a 4-week intervention delivered via a mobile app and wearable device (BioBase program) in comparison with a wait-list control group	262; university; 45.4%	Age: T at baseline- 19.9 years (SD 1.83, n=72), C at baseline- 19.84 (1.76, n=74); female: T at baseline- 62.5%, C at baseline- 63.5%; ethnicity: NR	RCT (T vs wait-list C)	Pre, 2 weeks, Post (4 weeks), 2-week FU (6 week); WEMWBS	BioBase: eclectic approach with mindfulness, biofeedback interventions, CBT^u^, and behavioral activation theory; well-being; via smartphone and wearable device; 4 weeks		Wait-list	*Perceived well-being:* group x time effect (post and FU); pre-post (within T) Cohen *d*=0.65, pre-FU (within T) Cohen *d*=1.16; *Conclusion:* intervention effective at improving well-being compared with wait-list control with sustained (2 week) effects at medium-large effect sizes	
Räsänen et al [50], 2016; Finland	Evaluate the efficacy of the web-based Student Compass program including 2 face-to-face meetings, tailored individual written feedback on the web, coping tools, and strategies	68; university; 87.9%	Age: 24.29 years (SD 3.28); female: 85.3%; ethnicity: NR	RCT (T vs wait-list C)	Pre, post, 12-month FU (intervention only); MHC-SF	ACT intervention; stress, anxiety, depression; guided, blended web-based and face-to-face; 7 weeks		Wait-list	*Psychological well-being:* group x time effect favoring T; significant improvement over time from baseline to 12 months FU in treatment group. Between pre-post corrected Cohen *d*=0.46; within pre-post corrected Cohen *d*=0.61; within pre-12- month FU corrected Cohen *d*=0.65; *Conclusion:* treatment more effective than wait-list control at improving psychological well-being with medium effect sizes	
Viskovich et al [44], 2018; Australia	Pilot-test a web-based ACT mental health promotion program called YOLO^v^ for university students	130; university; 40%	Age: 26.34 years (SD 7.96); female: 75.4%; White or Australian: 53.1%	Pilot RCT, 3 groups vary format: 1 (weekly flexible); 2 (full flexibility); 3 (sequential)	Pre, post; MHC-SF	YOLO ACT program; cognitive fusion, acceptance, mindfulness, values, and committed action; web-based; 4 weeks		2 Active: full flexibility delivery and sequential delivery	*Psychological well-being:* T significantly improved from preintervention to postintervention on the primary outcome of well-being across 3 delivery formats, Cohen *d*=0.25; *Conclusion:* intervention associated with improved well-being from pre to post time points with small effect size	
Viskovich et al [42], 2020; Australia	Evaluate the effectiveness of a web-based ACT mental health promotion intervention called YOLO in an RCT	2110; university; 29.3%	Age: 26.85 years (SD 8.77); female: 67.8%; ethnicity: NR	RCT (T vs wait-list C)	Pre, post; 12-week FU; MHC-SF	YOLO ACT program; cognitive fusion, acceptance, mindfulness, values, and committed action; web-based consisting of 4 modules; 4 weeks		Wait-list	*Psychological well-being:* time x condition effect favoring T Cohen *d*=0.37; *Conclusion:* intervention more effective than wait-list control at improving well-being with small effect size	

^a^Completers (%): percentage of participants who completed the postintervention data collection by the number of participants who were randomized to the intervention group.

^b^PWB: psychological well-being.

^c^T: treatment group.

^d^C: control group.

^e^NR: not reported.

^f^RCT: randomized controlled trial.

^g^TFS: The Flourishing Scale.

^h^Key outcomes and study conclusions italicized.

^i^ES: effect size.

^j^C-TAU: control-treatment as usual.

^k^MHC-SF: Mental Health Continuum−Short Form [53].

^l^mHealth: mobile health.

^m^IRR: incidence rate ratio.

^n^LYV: living your values.

^o^SPWB: Ryff Scales of Psychological Well-being-42 item [54].

^p^ACT: acceptance and commitment therapy.

^q^ACT-CL: ACT on college life.

^r^FU: follow-up.

^s^SBT: Stop, Breathe & Think.

^t^WEMWBS: Warwick-Edinburgh Mental Well-being Scale [55].

^u^CBT: cognitive behavioral therapy.

^v^YOLO: You Only Live Once.

### Mental Ill-Health Symptoms

The predominant measure of PWB was the Mental Health Continuum-Short Form [56] used in 62% (8/13) of the studies [39-44,49,50], followed by the scales of PWB [54] reported in 15% (2/13) of the studies [47,48], the Warwick-Edinburgh Mental Well-being Scale [55] reported in 15% (2/13) of the studies [38,45], and the Flourishing Scale [57] reported in 8% (1/13) of the studies [46]. A measure of the severity of mental health symptoms was included in 85% (11/13) of the studies and excluded in the other 15% (2/13) [45,47]. The Depression Anxiety Stress Scales (DASS) [30,58] were used in 31% (4/13) of studies [38,39,42,44], the Counseling Center Assessment of Psychological Symptoms was used in 23% (3/13) of studies [40,41,49], the Center for Epidemiological Studies–Depression [59,60] was used in 15% (2/13) of studies [46,48], and the Perceived Stress Scale [61] and the Hospital Anxiety and Depression Scale (HADS) [62] were used in 8% (1/13) of studies [43,50].

To better understand the prevalence and severity of mental ill-health symptoms in the general university student population, the DASS and HADS baseline scores were further analyzed using a software program that estimates the percentile scores and interval estimates for individual scores [63]. Of the studies that used these measures, some reported symptom cut-off scores as eligibility criteria [38,43] or targeted students who self-identified as distressed [50] or were actively seeking psychological treatment [41] and thus tended to report higher symptoms of mental ill-health. Studies using the DASS and HADS, which did not specify such eligibility criteria or targeted recruitment, may reflect the general mental health of university students. Levin et al [39] reported elevated depression (88th percentile, 95% CI 86-90) and anxiety (86th percentile, 95% CI 84-88) symptoms in university students compared with the general population. Viskovich et al [44] also reported elevated depression (86th percentile, 95% CI 84-88) and anxiety symptoms (90th percentile, 95% CI 88-92). At baseline, both the intervention and control groups reported by Viskovich et al [42] displayed elevated depression (intervention–93rd percentile, 95% CI 92-94; control 94th percentile, 95% CI 94-95), anxiety (intervention–94th percentile, 95% CI 93-95; control–94th percentile, 95% CI 93-95), and stress symptoms (intervention–96th percentile, 95% CI 95-97; control–97th percentile, 95% CI 96-98).

### Intervention Effectiveness

Of the 13 studies, 11 (85%) were eligible for inclusion in this meta-analysis [38-42,45,46,48-51]. Two corresponding authors were contacted and provided additional data needed for the meta-analysis, which were not reported in the published papers [39,51]. A study was excluded because the study design comprised a single-group pre-post comparisons [47]. In addition, a further study was excluded from the meta-analysis, as it reported on 3 intervention groups, each of which delivered identical content but in different formats (as planned, full flexibility and sequentially), and thus, it was not deemed to include an appropriate comparative group to address the core research question of this review [44].

**Figure 2:**
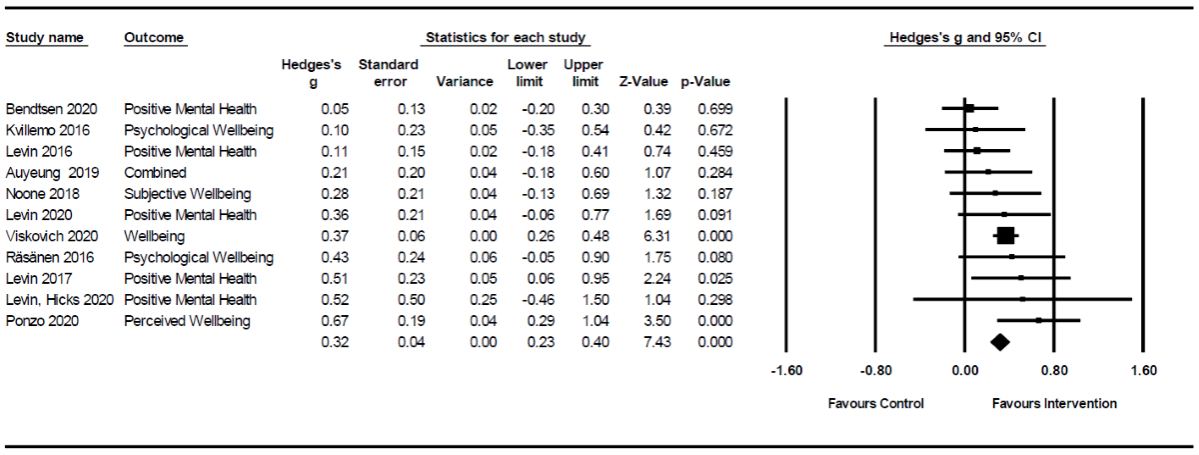
Effect of digital psychological interventions on psychological well-being in university student populations [38-42,45,46,48-51].

Of the 11 studies eligible for inclusion in the meta-analysis [38-42,45,46,48-51], the aggregate effect of treatment on PWB, as displayed in Figure 2, was small and statistically significant compared with controls (*k*=11; n=2903; Hedges *g*=0.32, 95% CI 0.23-0.4; *P<*.001). There was no evidence of significant heterogeneity (*Q_10_*=12.71; *P=*.24; *I*^2^=21.34; T^2^=0.01; *t*=0.08). Sensitivity analyses were performed to examine whether the treatment effect differed across studies with active (7/11, 64%) [38,39,45,46,48,50,51] and wait-list control (4/11, 36%) comparison groups [40-42,49]. When wait-list control studies were removed, the effect was reduced but remained statistically significant (Hedges *g*=0.22, 95% CI 0.08-0.35; *P=*.001).

All interventions were stand-alone, meaning they were offered to university students independent of university counseling services, and run by independent researchers or an independent youth mental health service such as Headspace [45]. Some studies used university services for recruitment, such as posting advertisements on existing student health care center websites [51] or waiting rooms [42], approaching students on wait-lists for counseling centers [40], or advertising through university student affairs [41]. Of the 13 studies included in the systematic review, acceptance and commitment therapy (ACT) was the most frequently adopted therapeutic orientation, present in 7 (54%) studies [39,41,42,44,47,49,50]. In addition, 23% (3/13) of studies adopted mindfulness interventions [40,45,48], 15% (2/13) used positive psychological interventions [43,46], and 8% (1/13) described an eclectic intervention [38]. A total of 46% (6/13) of interventions were web-based [39,42,44,47-49], 31% (4/13) were mobile apps [38,40,43,45], 15% (2/13) were available via mobile or desktop modes [41,46], and 8% (1/13) combined web-based and face-to-face modes of delivery [50]. Given the large number of ACT intervention studies, we conducted a subgroup analysis to evaluate the effectiveness of this therapeutic modality in relation to PWB. The meta-analytic effect of ACT-based treatments on PWB was small and statistically significant (Hedges *g*=0.35, 95% CI 0.25-0.45; *P<*.001).

### Risk of Bias

**Figure 3:**
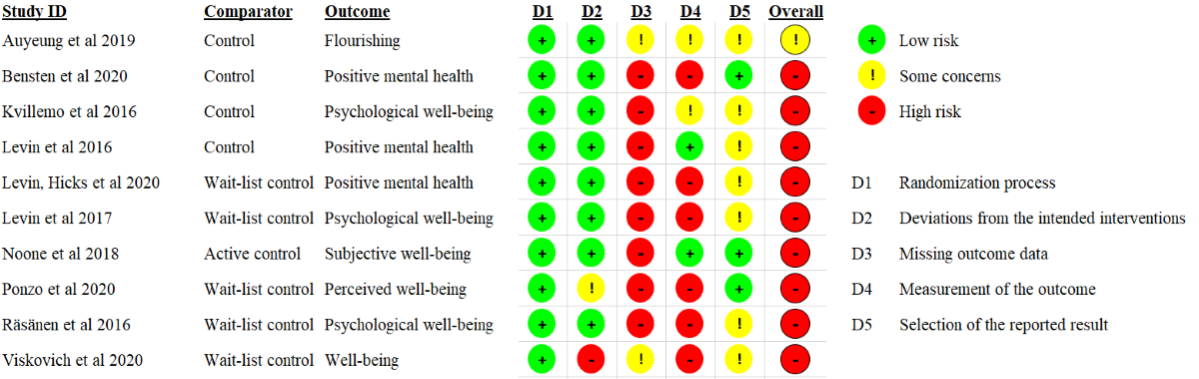
Risk of bias assessment using the Cochrane risk of bias tool (version 2) [38-42,45,46,48-51].

In relation to the study quality, based on study design, 77% (10/13) studies were rated according to the RoB-2 [33] and 23% (3/13) using the Downs and Black checklist [34]. As shown in Figure 3, overall, 8% (1/13) of studies were rated on the RoB-2 with “some concerns” of risk in relation to bias [46], and 69% (9/13) were rated as “high risk” of bias [38,39,41,42,45,48-51]. Missing outcome data were the most prevalent domain of concern, followed by the measurement of outcomes resulting from reliance on self-report measures. A total of 23% (3/13) of non-RCTs were rated using the Downs and Black checklist [34]. Each study was given a total score based on performance across 5 domains (reporting; external validity; internal validity-bias; internal validity-confounding; and power); each paper was scored (yes=1; no or unable to determine=0). Moreover, 15% (2/13) of studies were classified as overall fair quality [41,47] and 8% (1/13) as poor quality [44], according to classifications of the total score used in other reviews [64,65]. Common areas of weakness across 23% (3/13) of the studies assessed included a lack of reporting of adverse events, lack of attempt to blind participants or researchers, and not clearly reporting the intended analysis in methods, a priori. The overall estimation of the quality of evidence, based on the Grading of Recommendations Assessment, Development, and Evaluation assessment, was generally moderate (Multimedia Appendix 2).

A further RoB is study retention, which refers to the number of participants who completed the research study compared with those who discontinued the study or did not complete data collection at ≥1 time points. The most consistent retention data that could be collated across studies were the comparison of the number of participants randomized to the intervention with the number of participants who completed data collection at the post–time point for the specified primary outcome measures. The proportion of participants who completed postintervention data collection varied from 29.3% to 88.9% across the included studies, with an average of 63.6% completers across all studies (Table 1). Few studies have reported reasons for participant drop out, and the details of such reporting varied widely. In addition, most studies did not explicitly report adherence to the prescribed treatment. A study reported that although the reason for failing to complete the intervention was not systematically assessed, 9 participants emailed the coordinator and provided a reason, including lack of time, technical problems with software, or changed personal circumstances [45]. In relation to publication bias, visual inspection of the funnel plot suggested a largely symmetrical distribution, indicating a high likelihood that this review has captured all relevant studies and presents a low risk of publication bias for PWB (Multimedia Appendix 3). The Egger test of the intercept was not significant (intercept –0.09, SE 0.566; *P=*.44). Using the trim-and-fill method by Duval and Tweedie [66,67], only minor changes in values were observed, further supporting a low risk of publication bias.

## Discussion

### Principal Findings

This study aimed to synthesize the published literature on the effectiveness of digital mental health interventions for university students, specifically in relation to PWB. Our systematic review and meta-analysis found small to moderate effects on improving PWB in university students following exposure to a digital intervention. Beneficial effects remained when studies with a wait-list control group were removed, indicating that the effects of digital interventions remained robust in comparison with active controls. It is notable that ACT was the most prevalent theoretical framework underlying psychological interventions in the included studies. A subgroup analysis of 7 ACT-based interventions showed significant and small to moderate improvements in PWB. ACT targets transdiagnostic processes, such as the identification of values [68], which provide developmentally salient therapeutic targets for university students, while also accommodating heterogeneity in clinical needs. ACT as a therapeutic modality also coherently aligns with PWB as a key outcome, in addition to symptom alleviation [69-71]. Given that past reviews have identified PWB and flourishing as important mental health outcomes [17,18], especially for university students [19], the findings of this review provide further support for the development of digital psychological interventions grounded in ACT.

Our review resolves prior conflicting findings on the effectiveness of digital interventions for improving PWB in university students [23,24] and extends this work by exclusively focusing on PWB outcomes. Our findings partially align with the systematic review by Lattie et al [23], which identified 8 studies examining PWB as an outcome from digital mental health interventions for university students. The authors reported that 1 non-RCT and 6 RCTs found significant improvements using validated measures, whereas 1 study using qualitative measures did not find meaningful improvement. Our findings are in contrast with the meta-analysis conducted by Harrer et al [24], which found nonsignificant effects of digital interventions for university students on PWB across 4 RCTs. The specific focus of our review on PWB provides support for the capacity of digital interventions to meaningfully improve this specific psychological construct for university students, although these improvements were modest. Our review also identified ACT-based approaches as the most common therapeutic model used in the included studies, which also resulted in small to modest effects on PWB.

The baseline mental health data from the included studies indicated the presence of severe symptoms of mental ill-health in university student populations. Other studies have previously found that 20% to 31% of students experience a diagnoseable mental disorder [7,8]. The studies included in this review measured symptomology, not diagnosable mental health disorders, and found that depression symptoms in university students were more severe than that of 86% to 94% of the general population [39,42,44], anxiety symptoms were more severe than that of 86% to 94% of the general population [39,42,44], and stress symptoms more severe than that of 95% to 96% [42] of the general population. It is promising that this review found small to moderate and significant improvements in PWB as a result of digital interventions, despite the severity of symptoms. The recent COVID-19 pandemic is likely to place additional stressors on university students and exacerbate symptoms [72].

The COVID-19 global pandemic has had, and will continue to have, far-reaching consequences on individual, societal, and global functioning [73]. Although higher education rapidly pivoted to web-based learning in Western countries to reduce face-to-face contact and slow the spread of the virus [74], all 13 studies included in this review were conducted before the pandemic. It was noted during the literature search that many web-based psychological interventions were conducted for university students in 2020 and 2021; however, these were often excluded from this review because PWB was not an outcome. We argue that PWB is of even greater importance during a pandemic. The results of this review support the capacity of digital psychological interventions to improve PWB for university students; however, overall effect sizes were modest. Future research could target innovation to enhance the effectiveness of web-based interventions. Such innovation may include developing a better understanding of the predisposing and precipitating triggers of poor PWB among university students. Further innovation may also include identifying the active components of ACT approaches that address university students’ needs and consider approaches to integrate digital interventions within existing university-based care models.

Recent calls for the reform of university mental health services [11] are warranted. Duffy et al [11] recently emphasized the importance of integrated models of university-based mental health care that met the spectrum of student psychological needs [13] and embedded multidisciplinary mental health teams within campus services. Such models would comprehensively provide preventive and urgent treatment to high-risk populations that is likely to result in both immediate relief and long-term improvement in mental health trajectories [11]. In this review, all included studies featured stand-alone interventions, meaning they were not integrated with existing counseling or mental health support services; however, some studies used such services for recruitment purposes. For example, Levin et al [40] approached students on wait-lists for university counseling centers and provided a digital intervention while waiting for face-to-face appointments, effectively filling a gap in clinical need. In addition, Räsänen et al [50] reported that their web-based ACT intervention was also available more broadly in a Swedish university to 15,000 enrolled students in 2 formats: a self-help offering and twice a year offering in a coach-supported form. Firestone et al [47] concluded that such digital interventions could be integrated with university orientation programs in the future.

### Strengths and Limitations

This review provides a stronger evidence base to support the recommendations [11,13] for integrating effective digital interventions with existing counseling and student support services on university campuses, particularly when such interventions are grounded in an ACT therapeutic modality. This conclusion aligns with existing research that young adults are likely to endorse blended models of psychological care [75]. Future interventions could also consider the timing of such interventions; for example, before exams or during orientation may be when mental health needs are particularly salient to students. In addition, only 1 study included a 12-month follow-up after the intervention [50], highlighting the need for future research to ascertain whether the treatment effects of digital interventions persist over time for university students.

There are several methodological concerns regarding the quality of the available studies. Study retention is a commonly recognized challenge for digital psychological interventions [76,77], with a meta-analysis reporting dropouts from such programs ranging from 2% to 83%, with a weighted average of 31% [78]. In this review, we calculated study retention based on the proportion of participants who completed the postintervention data compared with those randomized or allocated to the intervention group. The proportion of completers varied from 29.3% to 88.9% across the included studies, with an average of 63.6% completing the digital intervention, similar to completion rates reported in previous reviews [73]. Future studies on digital interventions for university students should systematically collect both treatment adherence and study compliance data, accompanied by explicit reasons for drop out or discontinuation of the treatment.

In addition, a group of researchers conducted 3 of the included studies [39,40,49], potentially resulting in an undue influence of a paradigm or researcher’s approach or style of intervention. Given that 2 of these studies presented some of the strongest effect sizes for PWB [40,49], it may be that the effect was the result of something specific to this group’s implementation of ACT. Alternatively, a significant, positive finding in 2 independent studies by the same group of researchers may also increase confidence that results are less likely to have been a chance finding, assuming that bias was carefully managed. Further examination of ACT-based digital psychological interventions across different university student populations would clarify the effectiveness of these approaches. As discussed, the fail-safe N and funnel plot analyses suggest that there is a low risk of publication bias. Overall, the quality of the studies was rated as moderate, with the most common methodological issues potentially causing bias including failure to report complete outcome data and issues with the measurement of PWB. Future research should be strengthened by reporting greater detail when describing intervention content to facilitate an understanding of the mechanisms of change, consideration of dosage effects, and an assessment of the acceptability of such interventions.

### Conclusions

Overall, the results of this systematic review and meta-analysis indicate that digital psychological interventions are a promising area of research and clinical intervention for enhancing PWB among university students. The most common therapeutic modality for digital interventions was ACT, which theoretically focuses on strengthening the individual’s capacity to lead a rich and value-driven life, a goal which is consistent with PWB outcomes. The effect sizes demonstrated significant improvements in PWB for university students, albeit only modestly. The findings of this review encourage the further development of evidence-based digital interventions that target PWB in vulnerable populations such as university students.

## Appendix

Multimedia Appendix 1 Search strategy.

Multimedia Appendix 2 Grading of Recommendations Assessment, Development, and Evaluation quality assessment.

Multimedia Appendix 3 Funnel plot of studies included in the random-effects meta-analysis of digital psychological interventions for psychological well-being in university students compared with control groups.

## Abbreviations

ACT: acceptance and commitment therapy
DASS: Depression Anxiety Stress Scale
HADS: Hospital Anxiety and Depression Scale
PICO: population, intervention, control, and outcomes
PRISMA: Preferred Reporting Items for Systematic Reviews and Meta-Analyses
PWB: psychological well-being
RCT: randomized controlled trial
RoB: risk of bias
