# Supplementary material for: Digital Interventions for Psychological Well-being in University Students: Systematic Review and Meta-analysis
Source: J Med Internet Res. 2022 Sep 28;24(9):e39686. doi: 10.2196/39686 (PMC9557766; doi:10.2196/39686)
Supplement: Multimedia Appendix 1 [file jmir_v24i9e39686_app1.docx]

**Supplementary Materials A**

**Search Strategy**

**Search terms used across each database**

| **Population** | **Intervention** |
| --- | --- |
| **“University student*”** | **“Electronic Health Service*”** |
| **OR** | **OR** |
| **“Post graduate student*”** | **“Health Care Service*”** |
| **OR** | **OR** |
| **“College student*”** | **“Mobile Health”** |
| **OR** | **OR** |
| **“Undergraduate student*”** | **Telemedicine** |
|  | **OR** |
|  | **“Online Therap*”** |
|  | **OR** |
|  | **“Digital intervention*”** |
|  | **OR** |
|  | **Telepsychology** |
|  | **OR** |
|  | **Telehealth** |
|  | **OR** |
|  | **“Electronic health”** |
|  | **OR** |
|  | **Mhealth** |
|  | **OR** |
|  | **“Online consult*”** |
|  | **OR** |
|  | **“Online intervention”** |
|  | **OR** |
|  | **“Zoom consult*”** |
|  | **OR** |
|  | **“Computer intervention”** |
|  | **OR** |
|  | **Teleconsult*** |
|  | **OR** |
|  | **“Online Therap*”** |
|  | **OR** |
|  | **Ehealth** |

**EMBASE (Elsevier) Search Strategy**

| **1** | (("post graduate student*" or "college student*" or "undergraduate student*" or "university student*") and (telehealth or "electronic health service*" or "health care service*" or "mobile health" or telemedicine or "Online therap*" or "digital intervention*" or telepsychology or "electronic health" or Mhealth or "Online consult*" or "online intervention*" or "zoom consult*" or "computer intervention" or Teleconsult*)).af. |
| --- | --- |
| **2** | **limit 1 to (english language and yr="2000 -Current")** |

**MEDLINE (Ovid) Search Strategy**

| **Search Options** | **Actions** |
| --- | --- |
| ( “University student*” OR “Post graduate student*” OR “College student*” OR “Undergraduate student*” ) AND ( “Electronic Health Service*” OR “Health Care Service*” OR “Mobile Health” OR Telemedicine OR “Online Therap*” OR “Digital intervention*” OR Telepsychology OR Telehealth OR “Electronic health” OR Mhealth OR “Online consult*” OR “Online intervention” OR “Zoom consult*” OR “Computer intervention” OR Teleconsult* OR “Online Therap*” OR Ehealth ) | **Limiters** - Date of Publication: 20000101-20211231; English Language; Scholarly (Peer Reviewed) Journals  **Expanders** - Apply equivalent subjects  **Search modes** - Find all my search terms |

**PsycINFO (EBSCOhost) Search Strategy**

| **Search Options** | **Actions** |
| --- | --- |
| ( “University student*” OR “Post graduate student*” OR “College student*” OR “Undergraduate student*” ) AND ( “Electronic Health Service*” OR “Health Care Service*” OR “Mobile Health” OR Telemedicine OR “Online Therap*” OR “Digital intervention*” OR Telepsychology OR Telehealth OR “Electronic health” OR Mhealth OR “Online consult*” OR “Online intervention” OR “Zoom consult*” OR “Computer intervention” OR Teleconsult* OR “Online Therap*” OR Ehealth ) | **Limiters** - Peer Reviewed; Publication Year: 2000-; Published Date: 20000101-; English; Exclude Dissertations  **Expanders** - Apply equivalent subjects  **Search modes** - Find all my search terms |

**Web of Science (Thomson Reuters) Search Strategy**

| ((ALL=((“Electronic Health Service*” OR “Health Care Service*” OR “Mobile Health” OR Telemedicine OR “Online Therap*” OR “Digital intervention*” OR Telepsychology OR Telehealth OR “Electronic health” OR Mhealth OR “Online consult*” OR “Online intervention” OR “Zoom consult*” OR “Computer intervention” OR Teleconsult* OR “Online Therap*” OR Ehealth))) AND ALL=((“University student*” OR “Post graduate student*” OR “College student*” OR “Undergraduate student*”) )) AND (DT==("ARTICLE")) |
| --- |
