# Supplementary material for: Digital Interventions for Psychological Well-being in University Students: Systematic Review and Meta-analysis
Source: J Med Internet Res. 2022 Sep 28;24(9):e39686. doi: 10.2196/39686 (PMC9557766; doi:10.2196/39686)
Supplement: Multimedia Appendix 2 [file jmir_v24i9e39686_app2.docx]

**Supplementary Materials B**

**GRADE Quality Assessment**

**Author(s):** Gleeson, Ferrari

**Question:** Digital intervention compared to control groups for improving psychological wellbeing

**Setting:** University student populations

**Bibliography:**

| **Certainty assessment** | | | | | | | **№ of patients** | | **Effect** | | **Certainty** | **Importance** |
| --- | --- | --- | --- | --- | --- | --- | --- | --- | --- | --- | --- | --- |
| **№ of studies** | **Study design** | **Risk of bias** | **Inconsistency** | **Indirectness** | **Imprecision** | **Other considerations** | **Digital intervention** | **Control groups** | **Relative (95% CI)** | **Absolute (95% CI)** |  |  |
| **Psychological Wellbeing** | | | | | | | | | | | | |
| 10 | randomised trials | serious | not serious | not serious | not serious | none | 0/0 | 0/0 | not estimable |  | ⨁⨁⨁◯ Moderate |  |
| **Psychological Wellbeing** | | | | | | | | | | | | |
| 3 | observational studies | serious | not serious | not serious | not serious | none | 0/0 | 0/0 | not estimable |  | ⨁⨁⨁◯ Moderate |  |

**CI:** confidence interval

#### Explanations

a. The three non-randomised controlled trials were rated using the Downs and Black checklist. Each study was given a total score based on performance across five domains (reporting, external validity, internal validity – bias, internal validity - confounding, and power, each paper was scored (yes = 1, no or unable to determine = 0). Two studies were classified as overall fair quality, and one had poor quality, according to classifications of the total score used in other reviews. Common areas of weakness across the three studies assessed included a lack of reporting of adverse events, lack of attempt to blind participants or researchers and not clearly reporting intended analysis in methods, a priori.
