# Supplementary material for: Digital Interventions for Psychological Well-being in University Students: Systematic Review and Meta-analysis
Source: J Med Internet Res. 2022 Sep 28;24(9):e39686. doi: 10.2196/39686 (PMC9557766; doi:10.2196/39686)

**Supplementary Materials C**

Funnel plot of studies included in the random-effects meta-analysis of digital

psychological interventions for PWB in university students compared with control groups

[Grab your reader’s attention with a great quote from the document or use this space to emphasize a key point. To place this text box anywhere on the page, just drag it.]


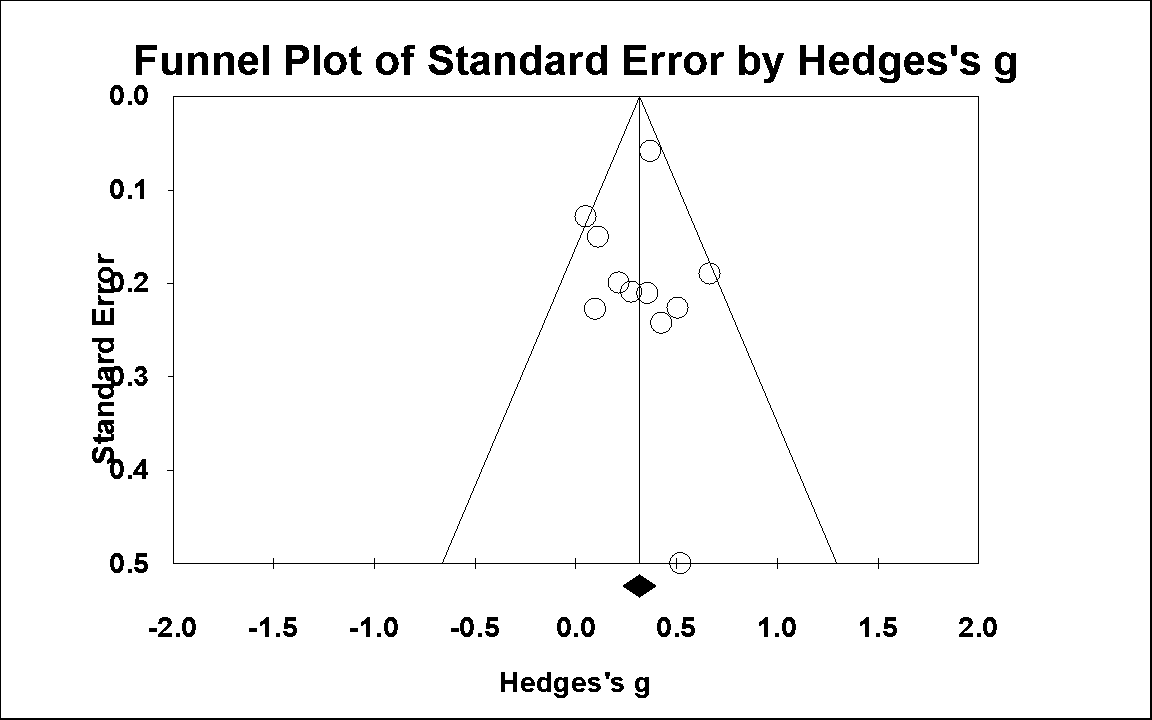

Supplement: Multimedia Appendix 3 [file jmir_v24i9e39686_app3.docx]
